# Successful Termination of a Large Aneurism, without Surgical Interference

**Published:** 1883-12

**Authors:** Henry Waldo

**Affiliations:** Physician to the Bristol Royal Infirmary


### SUCCESSFUL TERMINATION OF A LARGE ANEURISM, WITHOUT SURGICAL INTERFERENCE. By Henry Waldo, M.D., Physician to the Bristol Royal Infirmary.

Charles Lowman, a farm labourer, aged 40, admitted into Ward V. Bristol Royal Infirmary on 30th April, 1881. Eighteen months before admission, without any known cause—as he is not aware of having strained himself in any way, though constantly in the habit of lifting heavy weights into his cart,—the patient first felt pain in his left arm and hand, the fingers were numb and tingling. The pain, which was rheumatic in character, soon extended to the left shoulder and clavicle. This pain was much increased on making any exertion, and he was compelled to give up work. No previous diseases.

Upon admission there was an elongated pulsating swelling, which filled up the hollow between the left shoulder and the chest, its long axis lying parallel to the arm. In the neck it occupied the space between the anterior border of the trapezius and the sternal head of the sterno-mastoid, rising as high as the cricoid. Towards the front of the neck the tumour became deeper. It lay under the clavicle, which it raised a little. It extended down, and projected as a rounded pulsating swelling in the axilla. Its breadth was about three inches. There was fluctuation between its upper and lower limit, and there were some enlarged veins over it. There was a systolic murmur to be heard at the upper part of the tumour. The left chest was resonant up to edge of aneurism. Breath sounds feeble over posterior surface of chest, but equal. No spinal pain or tenderness. No dysphagia. Radial pulses equal. Respiration quiet. Pupils equal. Abdomen normal. Urine normal.

This was, no doubt, an aneurism of the left subclavian artery, with the upper part of the sac little thicker than a piece of blotting paper. I asked the surgeons to have a consultation upon the case, and they recommended that the man should be kept under chloroform while relays of students applied digital pressure on the proximal side of the aneurism.

The patient declined to have this done, and so the former treatment was continued. This consisted of perfect rest in bed. The left arm was "put up" as if a fractured clavicle existed. Low diet was ordered, and a restricted allowance of fluids.

Iodide of potassium was given in ten-grain doses three times a day, and this was gradually increased till at the end of seven weeks he was taking fifty grains three times a day. The improvement was very gradual. First, the pain lessened, and then disappeared. The part of the aneurismal sac that could be felt got thicker, and the tumour seemed to shrink. Three months after admission the patient told me that earlier in the day he felt "lost,"

and did not know where he was for one hour, and that the left leg, from the foot to the knee, seemed dead and tingled. After this attack I was unable to detect any pulsation in the aneurism, which felt quite solid, and there was no pulsation to be felt in the brachial, or radial, or any other artery in the left arm. The man left the Infirmary, 29th August, 1881, after a stay of four months. He has been doing his usual work ever since. I exhibited him to the Bath and Bristol Branch of the British Medical Association one year after his discharge, quite well.

